# Influence of Knowledge and Cultural Beliefs on Attitudes Toward HPV Vaccination Among Israeli Nurses and Nursing Students: Implications for Vaccine Advocacy

**DOI:** 10.3390/nursrep14040251

**Published:** 2024-11-11

**Authors:** Yulia Gendler, Nurit Ben-Aroya, Ayala Blau

**Affiliations:** 1 Department of Nursing, School of Health Sciences, Ariel University, Ariel 40700, Israel; ayalabl@ariel.ac.il; 2 Department of Infectious Diseases, Loewenstein Rehabilitation Hospital Center, Raanana 43100, Israel; nuritba3@clalit.org.il

**Keywords:** nursing, HPV vaccination, cultural beliefs, vaccine advocacy, nursing education, public health

## Abstract

Background: Human papillomavirus (HPV) poses a significant health burden, yet the vaccine which successfully prevents HPV and its associated cancers remains underutilized. Nurses play a crucial role in patient education and advocacy for HPV vaccination. This study explores how knowledge, along with cultural, religious, and social beliefs, shapes the attitudes of Israeli nurses and nursing students toward the HPV vaccine and their advocacy efforts. Methods: A cross-sectional study was conducted from August 2023 to January 2024 using an anonymous online questionnaire distributed via Facebook groups targeting Israeli nurses and nursing students. The questionnaire assessed demographic data, HPV-related knowledge, and attitudes toward HPV vaccination. Results: The study included 458 participants (229 nurses and 229 nursing students). Significant knowledge gaps were found, with 52% of participants incorrectly believing that the vaccine can cure existing infections and 47% mistakenly believing that it is administered in a single dose. Logistic regression revealed that nurses working in community settings were more likely to have positive attitudes toward HPV vaccination (OR = 2.98, 95% CI: 1.84–4.85). Higher levels of HPV-related knowledge (OR = 3.35, 95% CI: 2.10–5.35) and secular or traditional religious affiliations (OR = 2.45, 95% CI: 1.52–3.97) were strongly associated with positive attitudes toward and advocacy for the vaccine. Conclusions: Targeted educational programs addressing knowledge gaps, especially those tailored to Israel’s cultural and religious diversity, are crucial for empowering nurses and nursing students as advocates for HPV vaccination. Enhancing their understanding of HPV can increase vaccine uptake, reduce the incidence of HPV-related diseases, and strengthen public health initiatives in Israel.

## 1. Introduction

The human papillomavirus (HPV) is a group of more than 100 viral strains that infect the skin and mucous membranes. It is transmitted primarily through intimate contact and is the most prevalent sexually transmitted infection worldwide [1]. Typically asymptomatic, individuals often unknowingly transmit the virus to others. HPV can lead to various cancers, most notably cervical cancer, which is the second most common cancer in women after breast cancer. In 2018, cervical cancer caused more than 300,000 deaths globally, predominantly in low- and middle-income countries with limited access to public health services and early screening. In addition to cervical cancer, HPV is associated with other severe health issues, such as vaginal and vulvar cancers in women, penile cancer in men, and conditions such as anal and oropharyngeal cancers, genital warts, and recurrent respiratory papillomatosis in both genders [2,3]. Consequently, the economic burden of HPV in the US is substantial, estimated at approximately USD 49 million annually [4,5]. These epidemiological findings are consistent across developed nations, including Israel, where the prevalence of HPV-related morbidities is comparable and permeates all segments of the Israeli population [6].

The World Health Organization (WHO) aims to eliminate cervical cancer by 2030 through a strategy of vaccination, screening, and treatment. This includes ensuring that 90% of girls are fully vaccinated against HPV by age 15, 70% of women are screened by age 35 and again by age 45, and 90% of women with pre-cancerous lesions or invasive cancer receive appropriate treatment [7]. Vaccination has been proven to be an effective primary preventive measure against HPV. Currently, there are three types of HPV vaccines: Cervarix, Gardasil-4, and Gardasil-9 [8]. In 2013, Israel introduced a universal HPV vaccination program, initially targeting eighth-grade girls in middle schools and ninth-grade girls at health bureaus, with the Cervarix© vaccine added to the national health basket. In 2015, the program was expanded to include the Gardasil© vaccine for males aged 9 to 26. By the 2019–2020 school year, the Gardasil-9© vaccine was administered to both eighth-grade girls and boys as part of the national program, with catch-up vaccination available in the ninth grade and for individuals at risk up to age 26. Additionally, Gardasil-9 is offered in Israel to individuals up to age 45 [9,10].

Global HPV vaccination rates vary widely among countries and regions. In many high-income countries, HPV vaccination coverage ranges from 70% to over 90% among children and young adults (ages 9–26). However, in low- and middle-income countries, coverage rates can be significantly lower, often less than 50% [3,11]. In Israel, despite the vaccine’s safety, efficacy, availability, and inclusion in the health basket (which allows for free administration) and campaigns by the Ministry of Health and health maintenance organizations, HPV vaccination rates remain low, with annual rates fluctuating between 52% and 64% [12,13], in contrast to the high coverage rates (≥95%) for other routine vaccines (e.g., MMRV (measles, mumps, rubella, varicella) and Tdap (tetanus, diphtheria, pertussis) vaccines) offered through schools in Israel [14]. Low immunization rates against HPV are often attributed to inadequate information for informed decision-making, exposure to vaccine-hesitant misinformation online, and local structural barriers that hinder vaccine uptake. Additionally, concerns about discussing sexual health topics, particularly when they conflict with religious beliefs, further contribute to low vaccination rates [15,16].

Nurses, who constitute the largest segment of the healthcare workforce, are uniquely positioned to address the growing need for education about HPV and the benefits of HPV vaccines [17]. Their frequent and direct contact with patients, along with their primary role in patient education, enables them to help patients understand the risks and benefits of preventive treatment options. Patients often seek nurses for health-related advice, making them ideal primary contacts for immunization information, concerns, and adherence [18]. While their role as patient advocates is well established in Israel, their potential as HPV vaccine advocates is not yet fully recognized or widely embraced [10,13]. Given the authority and trust they command in healthcare delivery, nurses are ideally suited to advocate for HPV vaccination and significantly influence vaccine decisions [19,20].

In the United States, nurses are primary sources of guidance for recommended student vaccinations, with school nurses being particularly important in educating parents about HPV vaccination and advocating for cervical cancer prevention [19]. However, adequate knowledge levels are essential for nurses to effectively fulfill this role. Previous studies conducted in Cyprus [21] and Cameroon [22] have demonstrated that enhanced education and training for nurses and midwives can increase their willingness to recommend HPV vaccination, thus boosting uptake rates. Similarly, research from Turkey [23,24] and Spain [25] found that increasing nursing students’ knowledge of HPV can positively impact their intention to advocate for vaccination. In Israel, a recent study identified significant knowledge gaps among nurses regarding HPV and its vaccination, suggesting that, as in other countries, targeted education and training could be critical for improving vaccine advocacy and uptake [26].

Nurses and nursing students play a vital role in patient education, particularly in promoting vaccination. However, previous studies suggest that knowledge about HPV vaccination is lacking among both groups, and it remains unclear how these knowledge deficits impact their attitudes and willingness to advocate for HPV vaccination. Our study aims to assess the current knowledge and attitudes of Israeli nurses and nursing students towards HPV and its vaccine, identifying areas of misinformation. Additionally, this study aims to explore how cultural, religious, and social beliefs influence their willingness to advocate for HPV vaccination. By examining these factors, this research provides a deeper understanding of the unique influences shaping healthcare professionals’ support for HPV vaccination within the diverse Israeli population.

## 2. Methods

### 2.1. Setting

From August 2023 to January 2024, we conducted a cross-sectional study using an anonymous online questionnaire distributed via Facebook groups specifically targeted at nurses and nursing students. The questionnaire link, hosted on Qualtrics™, was shared in several professional groups that collectively reached thousands of potential participants across the country. These groups included members from diverse healthcare settings, such as hospitals, community clinics, and other healthcare facilities, ensuring a broad and representative sample of the nursing community. Respondents were invited to participate voluntarily, with no incentives provided, and were limited to one response per individual to maintain data integrity.

### 2.2. Participants

The study targeted Hebrew-speaking nurses and nursing students actively engaged in healthcare settings. All participants were required to be either currently practicing nurses or enrolled in nursing programs. No other exclusion criteria were applied, allowing for a wide range of experiences and backgrounds to be represented in the sample.

### 2.3. Ethical Considerations

The study protocol was reviewed and approved by the Institutional Review Board of Ariel University, approval code: AU-HEA-YG-20230111. Written informed consent was obtained electronically from all participants before they accessed the questionnaire. Participants were assured of their anonymity and the confidentiality of their responses.

### 2.4. Measures

The online questionnaire included three sections designed to assess the knowledge and attitudes of nurses and nursing students towards HPV and its associated vaccine:

Demographic data: Participants were asked to provide detailed demographic information, including age, gender, marital status, number of children, nationality, religiosity level, education, years of experience in nursing, workplace, role, and vaccination status against HPV.

Knowledge assessment: Participants’ knowledge about human papillomavirus (HPV) and its associated vaccine was assessed using an 18-item structured knowledge questionnaire that has been validated in previous studies. This tool was originally developed based on established literature and expert input, with its validity and reliability confirmed through prior research [27,28].

The questionnaire comprised a series of true/false statements designed to evaluate participants’ understanding of HPV transmission, the efficacy and purpose of the HPV vaccine, and common misconceptions about the virus and its vaccine. The participants were asked to indicate whether they believed each statement to be true or false, with an additional “don’t know” option for those who were unsure of the answer.

To assess the overall level of knowledge among participants, the percentage of correct, incorrect, and “don’t know” responses was calculated for each item. Higher percentages of correct answers indicated better knowledge, whereas higher percentages of incorrect or “don’t know” responses highlighted areas where misinformation or lack of knowledge was prevalent. The questionnaire addressed key topics, including whether HPV can cause serious health problems, the effectiveness of the HPV vaccine in preventing cervical cancer, and common myths such as HPV causing AIDS or genital herpes.

Attitude assessment: Attitude toward HPV vaccination was evaluated using a structured questionnaire specifically designed for this study. The questionnaire was divided into three sections: (1) attitudes toward the HPV vaccine and vaccination, (2) attitudes toward nurses’ role in HPV vaccination, and (3) attitudes toward the national vaccination program. Each section consisted of a series of statements to which participants responded via a 5-point Likert scale ranging from “strongly disagree” to “strongly agree”.

The first section, attitude towards the HPV vaccine and vaccination, comprised six items assessing participants’ beliefs about the importance and necessity of HPV vaccination for both men and women, the prevention of HPV-related diseases, and the justification for vaccination based on HPV’s association with cancer.

The second section, attitude towards nurses’ role in HPV vaccination, included four items evaluating participants’ views on the responsibility of nurses to recommend and advise on HPV vaccination, the importance of addressing negative opinions about the vaccine, and the perceived equivalence of the HPV vaccine with other routine vaccinations.

The third section, attitude towards the national vaccination program, consisted of two items measuring participants’ general support for routine vaccinations and their trust in the decisions made by health authorities, such as the Ministry of Health.

For items where agreement reflected a negative or incorrect attitude towards HPV vaccination, such as “women have already been vaccinated against HPV, so there is no need to vaccinate men as well”, reverse scoring was applied. This ensured that higher scores uniformly represented more positive or correct attitudes. The mean scores for each section were calculated, with reverse scoring applied as necessary, to provide an overall assessment of attitudes within each category, ensuring consistent interpretation across all items.

### 2.5. Data Analysis

To estimate the required sample size, we conducted a priori power analysis. Based on a 95% confidence level, a power (1-β) of 80%, and an effect size of 0.3, we determined that 82 participants were needed for each study group (nurses and nursing students). However, we recruited additional participants (229 per group) to allow for analysis of cultural subgroups in line with the aims of our study.

The data were extracted from Qualtrics™ and analyzed via IBM SPSS version 29. Descriptive statistics were calculated to summarize the demographic characteristics, knowledge levels, and attitudes of the participants. Frequencies and percentages were used for categorical variables, whereas means and standard deviations were reported for continuous variables. Independent samples *t* tests were conducted to compare attitudes between nurses and nursing students. A logistic regression model was used to identify factors predicting a positive attitude towards HPV vaccination, defined as a score of 3.5 or above on the attitude scale. The model included predictors such as gender, marital status, nationality, religiosity level, role (nurses vs. nursing students), workplace, years of experience in nursing, and knowledge level. Odds ratios (ORs) with 95% confidence intervals (CIs) were calculated to assess the strength of these associations.

## 3. Results

The study included 458 participants, consisting of 229 nurses and 229 nursing students. The average age was 47.5 years (SD = 10.2) for nurses and 26 years (SD = 4.8) for nursing students. Most participants were female (68.1% of nurses and 76.0% of nursing students) and married (82.0% of nurses and 61.1% of nursing students). In terms of nationality, 60.7% of nurses and 64.6% of nursing students identified as Jewish, while 21.8% of nurses and 31.4% of nursing students identified as Muslim Arabs.

For religiosity level, 36.2% of nurses and 32.7% of nursing students described themselves as secular. Additionally, 31.9% of nurses and 30.6% of nursing students identified as traditional, 28.4% of nurses and 35.4% of nursing students identified as religious, and 3.5% of nurses and 1.3% of nursing students described themselves as Ultra-Orthodox.

Regarding education, 39.3% of nurses held a Bachelor’s (BA) degree, and 10.7% held a Master’s (MA) degree, while all nursing students were enrolled in undergraduate nursing programs. Nurses reported an average of 13.5 years (SD = 13.0) of experience in the field, with 66.6% working in hospitals, 25.3% in community settings, and 8.1% in other roles. In terms of job roles, 40.4% of nurses were staff nurses, and 9.6% held managing positions.

For HPV vaccination, 21.0% of nurses and 37.1% of nursing students reported being vaccinated. Among those with children, 32.3% of nurses and 10.0% of nursing students reported their children were vaccinated. The sociodemographic characteristics of the study participants are presented in Table 1.

**Figure 1:**
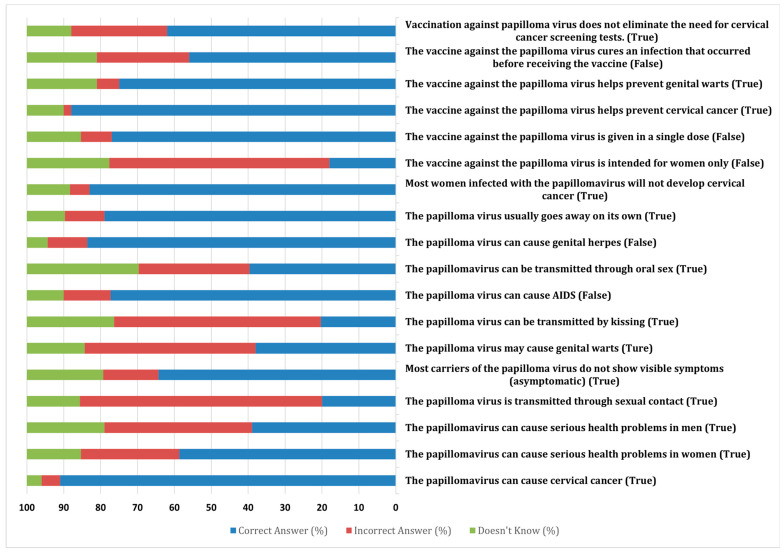
HPV and HPV vaccination knowledge assessment among nurses and nursing students.

The knowledge assessment (see Figure 1) revealed varying levels of understanding regarding HPV and its vaccine among participants. The majority of participants correctly identified that vaccination against the papillomavirus does not eliminate the need for regular screening (87%) and that the HPV vaccine helps prevent cervical cancer (92%). However, significant gaps in knowledge were observed in several areas. For example, 52% of participants incorrectly believed that the vaccine cures an existing HPV infection, and 47% mistakenly thought the vaccine is administered in a single dose. Additionally, misconceptions such as believing that the HPV vaccine is intended only for women (38%) and that HPV can cause genital herpes (31%) were prevalent. A notable proportion of participants (40%) were unsure of whether the papillomavirus can be transmitted through oral sex, and 29% mistakenly believed that HPV can cause AIDS. No significant difference was found between the levels of knowledge of nurses and those of nursing students.

The evaluation of attitudes toward HPV vaccination and the role of nurses highlighted both positive perceptions and potential areas for improvement among participants (see Table 2). Most participants demonstrated strong support for HPV vaccination, with nurses generally displaying a more favorable attitude than nursing students. For example, nurses were more likely to support vaccinating men to protect against HPV-related consequences, with a mean score of 4.6 ± 0.4 compared with 4.1 ± 0.2 for nursing students (*p* = 0.03). However, nursing students exhibited slightly more favorable attitudes towards the role of nurses in HPV vaccination, such as recommending the vaccine (mean score: 4.5 ± 0.4 for nursing students vs. 4.3 ± 0.5 for nurses) and addressing negative opinions about the vaccine (mean score: 4.4 ± 0.5 for nursing students vs. 4.2 ± 0.6 for nurses). Despite these differences, none were statistically significant, with *p* values of 0.27 and 0.24, respectively. Nurses expressed slightly higher trust in the national vaccination program, with a mean score of 4.5 ± 0.5 compared with 4.3 ± 0.6 for nursing students; however, this difference was not significant (*p* = 0.22). These findings suggest that while both groups generally hold positive attitudes, there are subtle variations, particularly in areas related to the role of nurses in HPV vaccination and trust in the national vaccination program. However, no significant differences were found between the overall attitudes of nurses and those of nursing students.

Several factors were found to predict a positive attitude toward HPV vaccination, as indicated by the logistic regression model (see Table 3). Participants who identified as secular or traditional were significantly more likely to have a positive attitude towards HPV vaccination compared to those who identified as religious or Ultra-Orthodox Jewish (OR = 2.45, 95% CI: 1.52–3.97, *p* < 0.001). Additionally, those working in community settings demonstrated a higher likelihood of having a positive attitude than those in other workplaces (OR = 2.98, 95% CI: 1.84–4.85, *p* < 0.001). Higher levels of knowledge about HPV and its vaccine were also strongly associated with a positive attitude (OR = 3.35, 95% CI: 2.10–5.35, *p* < 0.001).

Factors such as gender, marital status, nationality, and years of experience in nursing were not significantly associated with positive attitudes towards HPV vaccination, although some trends were observed. Specifically, participants from non-Jewish backgrounds showed a slight, non-significant decrease in positive attitudes compared with Jewish participants. Additionally, those with more years of nursing experience were slightly more likely to have a positive attitude towards HPV vaccination.

## 4. Discussion

The findings from this study provide valuable insights into the knowledge and attitudes of Israeli nurses and nursing students towards HPV and its associated vaccine While nurses play a critical role in patient education and vaccine advocacy, our results suggest areas where knowledge may be limited, potentially affecting their ability to effectively promote HPV vaccination. Specifically, misconceptions were identified regarding the vaccine’s ability to cure existing HPV infections, the belief that the vaccine is administered in a single dose, and confusion about the full scope of the impact of HPV, including its transmission and the range of cancers it can cause.

These findings are consistent with those of previous studies conducted in various countries, such as the USA, the UK, and China, where knowledge deficits among nurses were similarly noted. In the USA, school nurses have been increasingly recognized as pivotal in the HPV immunization campaign, emphasizing the importance of their role in educating youth about the vaccine’s benefits [29]. However, similar to our findings, studies in other regions have highlighted the need for improved literacy among nurses concerning HPV-related issues. In the, research revealed that while primary care practice nurses generally possessed adequate knowledge, a significant minority still harbored fundamental misconceptions about HPV and its vaccine [30]. This is echoed by findings in China, where a substantial proportion of nurses were unaware of critical aspects of HPV and its potential consequences [31].

The study by Runngren et al. (2022) on Swedish school nurses provides further context for these findings by exploring the experiences of nurses who are directly involved in administering HPV vaccines. The study highlights the delicate balance these nurses must strike between maintaining a neutral stance and actively promoting the vaccine to increase uptake. This balance is often influenced by nurses’ own knowledge and the official guidelines they follow. The study also underscores the ethical dilemmas nurses face, particularly when their professional obligation to increase vaccination rates conflicts with the need to respect patient autonomy. These dilemmas can be exacerbated by a lack of clear guidelines and adequate training, which can lead to inconsistencies in how nurses communicate the importance of the HPV vaccine to their patients [32].

Our study reveals the significant impact of cultural, religious, and social beliefs on attitudes towards HPV vaccination, with secular or traditional participants showing more positive attitudes compared to those identifying as religious or Jewish Ultra-Orthodox. This finding aligns with broader research indicating that such beliefs can heavily influence health behaviors, including vaccination [33]. In religious communities, concerns about the vaccine’s association with sexual activity may lead to hesitancy or refusal, driven by fears that it could be perceived as endorsing premarital sexual activity, conflicting with moral or religious values [34,35]. In a multicultural and religiously diverse society such as Israel, these insights highlight the need for culturally sensitive public health campaigns that are carefully tailored to address the specific concerns and beliefs of different communities to effectively promote HPV vaccination [27].

Moreover, the study identified a significant association between workplace setting and attitudes towards HPV vaccination. Participants working in community settings were more likely to have positive attitudes than those in other workplaces. This likely reflects greater exposure to public health initiatives and a stronger emphasis on preventive care within community health settings. Previous studies have suggested that community-based nurses may be more attuned to the importance of vaccination and preventive measures, which could explain their more favorable attitudes [36,37].

The logistic regression analysis further demonstrated that higher levels of knowledge about HPV and its vaccine are strongly associated with a positive attitude towards HPV vaccination, a finding that is consistent with previous research [30]. Studies across various healthcare settings have repeatedly shown that increased knowledge among healthcare providers is linked to more proactive health behaviors, including vaccine advocacy [38,39]. This alignment underscores the critical role of education in shaping healthcare professionals’ attitudes and behaviors. These findings suggest that targeted educational programs designed to enhance HPV-related knowledge among nurses and nursing students could significantly improve their effectiveness as advocates for vaccination. By equipping these healthcare providers with accurate information and a deeper understanding of HPV, public health initiatives aimed at increasing vaccine uptake could be more successful, ultimately contributing to a reduction in HPV-related diseases.

### 4.1. Limitations

While this study provides important insights, it is essential to acknowledge several limitations. First, the study relied on self-reported data, which may be subject to social desirability bias, where participants might respond in a manner that they believe is expected rather than expressing their true beliefs or practices. Second, the cross-sectional design of the study limits our ability to draw causal inferences, capturing attitudes and knowledge at only one point in time. Additionally, the use of convenience sampling via Facebook groups may not fully represent the broader population of Israeli nurses and nursing students, as it only includes those who own and regularly use Facebook, potentially limiting the generalizability of the findings. Furthermore, as the study did not collect information on the specific years of study, potential differences in knowledge levels across various study years among nursing students could not be assessed. This limitation highlights the need for further research to explore these variations in knowledge levels.

### 4.2. Implications for Practice and Future Research

The findings of this study have significant implications for public health practice in Israel. Targeted educational programs that address the specific knowledge gaps identified could enhance the role of nurses and nursing students as advocates for HPV vaccination. Such programs should be culturally sensitive and consider the diverse religious and social backgrounds of the population. Moreover, ongoing research is needed to explore the long-term impact of improved education on vaccination rates and to assess the effectiveness of different educational interventions.

Future research should also focus on longitudinal studies to track changes in knowledge and attitudes over time, particularly as nurses and nursing students are exposed to new information and public health campaigns. Additionally, exploring the impact of specific cultural and religious beliefs on vaccination attitudes could provide deeper insights into how best to tailor public health messages in multicultural societies such as Israel.

### 4.3. Conclusions

To enhance the effectiveness of nurses and nursing students as advocates for HPV vaccination in Israel, it is crucial to implement targeted educational programs that address the specific knowledge gaps identified in this study. These programs should be designed with cultural sensitivity, taking into account the diverse religious and social backgrounds of the population to ensure that they resonate with all communities. By improving healthcare providers’ knowledge of HPV and its vaccine, these initiatives can empower them to advocate more effectively for vaccination, ultimately increasing vaccine uptake and reducing the incidence of HPV-related diseases. Additionally, public health campaigns should focus on clear communication strategies that correct common misconceptions and emphasize the importance of preventive care, particularly in community settings where nurses are more frequently involved in public health initiatives. These practical steps can significantly strengthen the role of nurses in promoting HPV vaccination and contribute to better health outcomes across Israel.

## Figures and Tables

**Table 1:** Demographic characteristics and descriptive information (n = 458).

Variable	Categories	n (%) or Mean (SD)	
		Nurses (n = 229)	Nursing Students (n = 229)
Age		47.5 (10.2)	26 (4.8)
Gender	Male	73 (31.9)	55 (24.0)
	Female	156 (68.1)	174 (76.0)
Marital status	Single/Other	15 (6.6)	85 (37.1)
	Married	188 (82.0)	140 (61.1)
	Widowed	11 (4.8)	0
	Divorced	15 (6.6)	4 (1.8)
Number of children	0	15 (6.6)	97 (42.4)
	1–2	53 (23.1)	107 (46.7)
	3–4	63 (27.5)	25 (10.9)
	5 and above	98 (42.8)	0
Nationality	Jewish	139 (60.7)	148 (64.6)
	Muslim Arab	50 (21.8)	72 (31.4)
	Christian (all)	20 (8.8)	9 (4.0)
	Bedouin	14 (6.1)	0
	Druze	6 (2.6)	0
Religiosity level	Secular	83 (36.2)	75 (32.7)
	Traditional	73 (31.9)	70 (30.6)
	Religious	65 (28.4)	81 (35.4)
	Ultra-Orthodox	8 (3.5)	3 (1.3)
Education	BA	180 (39.3)	n/a
	MA	49 (10.7)	n/a
	Nursing student	n/a	229 (100)
Years of experience in nursing		13.5 (13.0)	n/a
Workplace	Community	116 (25.3)	n/a
	Hospital	305 (66.6)	n/a
	Other	37 (8.1)	n/a
Role	Staff nurse	185 (40.4)	n/a
	Managing position nurse	44 (9.6)	n/a
	Nursing student	n/a	229 (100)
Vaccinated against HPV	No	181 (79.0)	144 (62.9)
	Yes	48 (21.0)	85 (37.1)
Children vaccinated against HPV	No	103 (45.0)	20 (8.8)
	Yes #	74 (32.3)	23 (10.0)
	n/a *	52 (22.7)	186 (81.2)

Footnotes: # Out of 300 nurses and nursing students with children eligible for HPV vaccination, 97 (32.3%) had their children vaccinated against HPV. * Has no children or all children are below the age of eligibility for HPV vaccination.

**Table 2:** Comparison of attitudes towards HPV vaccination between nurses and nursing students.

Attitude Item	Nurses (n = 229)	Nursing Students (n = 229)	*p*-Value
1. Attitude towards HPV vaccine and vaccination	4.2 ± 0.5	3.8 ± 0.4	0.04
Men should be vaccinated to protect partners from cervical cancer and other HPV consequences	4.6 ± 0.4	4.1 ± 0.2	0.03
No need to vaccinate men if women are already vaccinated (reversed)	3.9 ± 0.5	3.8 ± 0.8	0.13
Important to vaccinate women against HPV to prevent anal/genital warts	4.5 ± 0.5	4.0 ± 0.5	0.04
HPV does not cause enough cancer cases to justify vaccination (reversed)	3.7 ± 0.6	3.2 ± 0.4	0.05
No sense in vaccinating women; other treatments for warts exist (reversed)	4.0 ± 0.6	3.6 ± 0.4	0.04
Important to vaccinate women to prevent men from contracting HPV	4.4 ± 0.5	4.0 ± 0.6	0.02
2. Attitude towards nurses’ role in HPV vaccination	4.3 ± 0.5	4.5 ± 0.4	0.25
Recommend getting vaccinated when consulted about HPV vaccine	4.3 ± 0.5	4.5 ± 0.4	0.27
Address negative opinions about HPV vaccine with knowledge and explanation	4.2 ± 0.6	4.4 ± 0.5	0.24
Part of nurse’s responsibility to advise patients about the HPV vaccine	4.4 ± 0.5	4.6 ± 0.4	0.21
HPV vaccine is as important as other routine vaccinations	4.3 ± 0.5	4.5 ± 0.4	0.26
3. Attitude towards national vaccination program	4.6 ± 0.5	4.4 ± 0.6	0.20
Belief in the importance of all routine vaccinations, including booster doses	4.7 ± 0.4	4.5 ± 0.5	0.18
Trust in the decisions made by Ministry of Health officials	4.5 ± 0.5	4.3 ± 0.6	0.22

Footnotes: Items marked as “reversed” were reverse scored. In these items, agreement indicates a negative or incorrect attitude towards HPV vaccination, so the scale is inverted to ensure that higher scores uniformly reflect more positive or correct attitudes. For example, on a 5-point Likert scale, a score of 5 becomes 1, 4 becomes 2, and so on, before the mean is calculated.

**Table 3:** Factors predicting positive attitude towards HPV vaccination—a logistic regression model.

Factors	OR	95% CI	*p*-Value
Gender			
Male (Ref.)	1 (Ref.)		
Female	1.15	0.68–1.94	0.61
Marital Status			
Single (Ref.)	1 (Ref.)		
Others (married, widowed, divorced)	1.08	0.66–1.77	0.72
Years of experience in nursing (per additional year)	1.02	0.98–1.06	0.89
Nationality			
Jewish (Ref.)	1 (Ref.)		
Others (Muslim-Arab, Christian, Bedouin, Druze)	0.87	0.54–1.38	0.55
Religiosity Level			
Religious and Jewish Ultra-Orthodox (Ref.)	1 (Ref.)		
Others (secular, traditional, religious)	2.45	1.52–3.97	<0.001
Role			
Nurses (Ref.)	1 (Ref.)		
Nursing Students	1.12	0.73–1.72	0.48
Workplace			
Others (Ref.)	1 (Ref.)		
Community	2.98	1.84–4.85	<0.001
Knowledge Level			
Low (Ref.)	1 (Ref.)		
High	3.35	2.10–5.35	<0.001

Footnotes: 1. Positive attitude definition: A positive attitude towards HPV vaccination is defined as a score of 3.5 or above on the attitude scale. 2. Knowledge level calculation: High knowledge level: Participants who correctly answered at least 14 (77.8%) of the knowledge-assessment questions. Low knowledge level: Participants who correctly answered fewer than 77.8% of the knowledge-assessment questions.

## Data Availability

The data supporting the findings of this study are included within the paper.

## Public Involvement Statement

No public involvement in any aspect of this research.

## Guidelines and Standards Statement

This manuscript was drafted against the STROBE guidelines for cross-sectional studies.
